# Natural Products in Modern Biology: Ancient Wisdom for Today’s Challenges

**DOI:** 10.3390/biology10050369

**Published:** 2021-04-25

**Authors:** Marcus Krüger, Peter Richter, Sebastian M. Strauch

**Affiliations:** 1 Cell and Tumor Biology Group, Department of Microgravity and Translational Regenerative Medicine, Otto von Guericke University, 39106 Magdeburg, Germany; 2 Gravitational Biology Group, Cell Biology Division, Department of Biology, Friedrich-Alexander University, 91058 Erlangen, Germany; peter.richter@fau.de; 3 Postgraduate Program in Health and Environment, University of Joinville Region, 89219-710 Joinville, SC, Brazil; sebastian.michael@univille.br

Nature provides a unique diversity of primary and secondary metabolites. Today, more than 325,000 compounds have been identified [1] and the number keeps increasing. Natural products have been used since ancient times and have often been the sole means to treat diseases and injuries. Even our earliest ancestors chewed on herbs to relieve pain or wrapped leaves around wounds to improve healing. During the past decades, after technological and scientific developments allowed researchers to better understand the biological effects of drugs on cells, organisms, and the human body, natural products played a key role in drug discovery, especially for cancer and infectious diseases [2,3]. The discovery of penicillin by Alexander Fleming, or the discovery of streptomycin by Selman A. Waksman, were not only history-changing milestones that greatly affected human life, but also heralded a ‘Golden Age’ of drug discovery based on natural compounds. Especially in the past few years, there is a renewed interest in the use of natural products in (complementary) medicine and, more importantly, their role as a basis for drug development. These molecules have evolved their function over millions of years, making them often more efficient than synthetic substances if a specific biological activity is needed.

In this Special Issue we summarized the most recent findings and developments in natural product-based drug discovery:

**Antimicrobials:** Antibiotics have highly increased human life expectancy. However, multidrug-resistant pathogens have become a common problem and conventional antibiotics are becoming increasingly ineffective due to their overuse and abuse for a long time [4]. Amphibian skin secretions are remarkable sources of novel bioactive peptides. Wang et al. [5] described the peptide kassinatuerin-3 isolated from skin secretions of the African frog *Kassina senegalensis*. It showed antimicrobial activity against Gram-positive bacteria and was also active in *S. aureus* biofilm eradication. In order to generate peptides with specific antimicrobial activity, He et al. [6] focused on the structure–activity relationship and designed a novel peptide based on brevinin-1 (from skin secretions of *Lithobates palustris*).

**Antidiabeticals:** Diabetes has become a major public health issue approaching epidemic proportions globally [7]. Most modern drugs that control and lower blood glucose levels have many side effects and cause some serious medical problems during treatment. Therefore, alternative medicines are getting more and more interesting. Tran et al. [8] reviewed active compounds and pharmacological effects of some popular medical plants which have been widely used in diabetic treatment. Draganescu et al. [9] found antidiabetic effects of present lignans and polyphenols from flaxseed extract on streptozotocin-induced diabetic rats. Flaxseeds play an important role in human health because their oil contains essential fatty acids, such as α-linolenic acid and linoleic acid. The study by Draganescu and coworkers indicated that the consumption of polyphenol and lignan compounds could have therapeutic potential in diabetes management [9]. Maher et al. [10] reported on the discovery of specific antidiabetic compounds from *Withania coagulans*. The related species *W. somnifera*, known in India as Asghawhanda, is used traditionally to treat different pathologies including diabetes [11].

**Anti-inflammatory drugs:** Many inflammatory diseases are becoming common in today’s aging society [12]. Side effects and high costs are often the disadvantages of clinically used anti-inflammatory drugs. Abdallah et al. [13] demonstrated for the first time the anti-ulcer effect of *Acacia saligna* in the rat model of acetic-acid induced ulcerative colitis. The butanol extract of *A. saligna* and its nanoformulations enhanced this effect and was comparable to the reference drug dexamethasone. Martins et al. [14] evaluated the anti-inflammatory effect of *Croton rhamnifolioides* essential oil complexed in cyclodextrin. The complex showed improved anti-inflammatory activity in mice models of acute and chronic inflammation. The authors believe that the complexation with cyclodextrin can improve the pharmacological effects of essential oils and may also contribute to a reduced incidence of toxic effects.

**Epigeneticals:** Histone deacetylases are key components of the epigenetic machinery that controls gene expression. These enzymes are not only responsible for the maintenance of fundamental cellular processes but are also involved in serious human diseases such as cancer [15]. For this Special Issue, Luparello et al. [16] summarized studies on marine invertebrate-derived compounds that possess inhibitory properties on histone deacetylases and grouped the producing species according to their taxonomic hierarchy. Particularly, marine habitats represent an eminent source of disparate bioactive secondary metabolites produced by bacteria, plants and animals, whose number is expected to increase rapidly [17].

Innovative strategies in drug discovery are of great importance to meet today’s global health and environmental challenges, including the rise of drug-resistant pathogens, increasing cancer incidence or the growing prevalence of other diseases such as diabetes. Driven by recent developments, the interest in natural products as drugs is growing again. Fortunately, only a very small part of this ‘gold mine’ has been discovered yet. In addition, some of the known compounds have only been structurally described but never evaluated biologically. We would like to thank all the authors who contributed to this Special Issue. Continuing insights both into natural product biology and into the molecular basis of various diseases will reveal more promising applications for future therapies with natural products.
